# The Effect of Athletes’ Probiotic Intake May Depend on Protein and Dietary Fiber Intake

**DOI:** 10.3390/nu12102947

**Published:** 2020-09-25

**Authors:** Joy Son, Lae-Guen Jang, Byung-Yong Kim, Sunghee Lee, Hyon Park

**Affiliations:** 1 Exercise Nutrition & Biochemistry Lab., KyungHee University, Yongin 17104, Korea; joy_son@khu.ac.kr (J.S.); francisjang@yonsei.ac.kr (L.-G.J.); 2 ChunLab, Inc., Seocho-gu, Seoul 06725, Korea; bykim@chunlab.com; 3 Research Lab., Ildong Pharmaceutical Co., Ltd., Hwaseong 18449, Korea; slee@ildong.com

**Keywords:** gut microbiota, athletes, bodybuilder, protein, dietary fiber, probiotic

## Abstract

Studies investigating exercise-induced gut microbiota have reported that people who exercise regularly have a healthy gut microbial environment compared with sedentary individuals. In contrast, recent studies have shown that high protein intake without dietary fiber not only offsets the positive effect of exercise on gut microbiota but also significantly lowers the relative abundance of beneficial bacteria. In this study, to resolve this conundrum and find the root cause, we decided to narrow down subjects according to diet. Almost all of the studies had subjects on an ad libitum diet, however, we wanted subjects on a simplified diet. Bodybuilders who consumed an extremely high-protein/low-carbohydrate diet were randomly assigned to a probiotics intake group (*n* = 8) and a placebo group (*n* = 7) to find the intervention effect. Probiotics, comprising *Lactobacillus acidophilus*, *L. casei*, *L. helveticus*, and *Bifidobacterium bifidum,* were consumed for 60 days. As a result, supplement intake did not lead to a positive effect on the gut microbial environment or concentration of short-chain fatty acids (SCFAs). It has been shown that probiotic intake is not as effective as ergogenic aids for athletes such as bodybuilders with extreme dietary regimens, especially protein and dietary fiber. To clarify the influence of nutrition-related factors that affect the gut microbial environment, we divided the bodybuilders (*n* = 28) into groups according to their protein and dietary fiber intake and compared their gut microbial environment with that of sedentary male subjects (*n* = 15). Based on sedentary Korean recommended dietary allowance (KRDA), the bodybuilders′ intake of protein and dietary fiber was categorized into low, proper, and excessive groups, as follows: high-protein/restricted dietary fiber (*n* = 12), high-protein/adequate dietary fiber (*n* = 10), or adequate protein/restricted dietary fiber (*n* = 6). We found no significant differences in gut microbial diversity or beneficial bacteria between the high-protein/restricted dietary fiber and the healthy sedentary groups. However, when either protein or dietary fiber intake met the KRDA, gut microbial diversity and the relative abundance of beneficial bacteria showed significant differences to those of healthy sedentary subjects. These results suggest that the positive effect of exercise on gut microbiota is dependent on protein and dietary fiber intake. The results also suggest that the question of adequate nutrition should be addressed before supplementation with probiotics to derive complete benefits from the intervention.

## 1. Introduction

Adequate nutritional intake, along with exercise, not only restores energy stores depleted by exercise-induced fatigue but also enhances overall fitness [1,2,3]. Among the various effects of exercise, the importance of altered gut microbiota has become increasingly evident because of the study of the human microbiome and the emergence of various tools for genomic analysis [4,5]. Recent studies have reported that factors underlying host fitness are also associated with gut microbial status and regulation [6,7,8,9]. For example, it has been reported that the effects of exercise on diabetes [10], obesity [11], and cardiovascular health [12] are mediated via changes in the gut microbial environment, such as increased diversity and the presence of beneficial bacteria. In addition, a recent study by Scheiman et al. reported that the gut microbiome of marathon runners is richer in the genus *Veillonella* than that of the control group, and *V. atypica* has been shown to improve athletes′ recovery and performance via lactic acid metabolism [13]. Lactic acid is rapidly formed in muscles during high-strength aerobic exercise to produce propionate. Similarly, exercise-induced changes in the microbial community affect energy metabolism, immune function, and oxidative stress in athletes, thereby influencing various bodily functions [14].

Studies on probiotics have been devoted to improving disease status or obesity [4,11,15]. The correlation between obesity-related diseases and changes in the gut microbiome is well-established [4,11,15], but relatively few studies have explored the relationship between motor stimulation, microbial diversity, and sedentary lifestyles [16,17]. A recent study by Jang et al. that targeted bodybuilders, long-distance runners and sedentary people showed that high protein consumption combined with low dietary fiber intake offsets exercise-induced improvements in the gut microbial environment. In addition, the relative abundance of short-chain fatty acid (SCFA)-producing bacteria was significantly lower in bodybuilders with significantly higher protein intake than in sedentary subjects and middle- to long-distance runners [18]. However, in addition to these promising quantitative indicators, gut mucosal colonization, which is directly induced by probiotic ingestion, is not uniform, and the effectiveness of various probiotics is disputed [19].

Several in vitro studies have reported selective colonization of the intestinal epithelium by probiotic-derived microorganisms. This makes it difficult to analyze the effects of probiotics on athletes′ gut microbiota and SCFAs. To overcome these limitations, and to recruit subjects that are more relevant to our aims, we needed to narrow our prospective pool of subjects. We assumed that probiotics may have differential effects in those who eat a specific diet vs. those who eat more freely. With regards to diet, bodybuilders were more suitable than those who practice other sports. Therefore, we attempted to identify specific factors that affect the microbial community of bodybuilders during probiotic intake and investigated whether probiotic intake may supplement the effects of exercise on gut microbial status, as reported in many previous studies. We aimed to reach conclusions regarding the correlations between exercise, probiotic intake, and dietary status.

## 2. Materials and Methods

### 2.1. Study Design

See Figure 1.

### 2.2. Recruitment of Participants and Supplementary Information

Bodybuilders who agreed to take probiotics were recruited for this study. Those who had been exposed to antibiotics within 6 months or had immune, digestive, acute or chronic cardiovascular disease and metabolic disorders were excluded.

A total of 20 subjects who met the enrollment criteria were randomly assigned to the probiotic group (*n* = 10) or the placebo group (*n* = 10). After providing written informed consent (KHSIRB 2016-011, KHSIRB-17-036), the volunteers received either a probiotic supplement or a similar-looking placebo (Ildong Pharmaceutical Co., Republic of Korea) for 60 days. The results of the random assignment are presented in Table S1.

- Probiotic group: Capsule consisting of 10^12^ CFU of each of the following species: *L. acidophilus, L. casei, L. helveticus,* and *Bifidobacterium bifidum*;
- Placebo group: Capsule consisting of corn starch.

The selected bacteria were associated with exercise based on differences at the genus level during gut microbiome analysis according to nutritional intake; 10 strains were used (Figure S1), which are commonly used in the preparation of probiotics [20,21,22,23,24]. Probiotics, which had proven stability in animal experiments, were provided to the participants in the form of capsules. The placebo group received capsules filled with corn starch with similar shape, size, and color as the probiotic capsules, and the two were indistinguishable by the naked eye. Probiotics or placebo were ingested once a day for 60 days.

During the 60-day intervention period, consent was provided by all subjects, except for four subjects (two in the probiotics group/two in the placebo group) who abandoned the study prior to pre-intervention collection and one subject (one in the placebo group) who dropped out immediately prior to post-intervention collection.

Table 1 shows the characteristics of the subjects in the probiotic group (*n* = 8) and the placebo group (*n* = 7), excluding the five subjects who dropped out.

The subjects were periodically monitored to ensure that nutritional intake was not altered during the supplement intake period, which occurred during peak bodybuilding season; the researchers contacted the subjects each week to confirm that they had not begun a special or unusual diet. There was no significant difference in the characteristics of the study subjects before and after the probiotic intake period.

After the intervention experiment (part 1), to clarify the influence of nutrition-related factors, we decided to combine the pre-intervention data (*n* = 16) with data from Jang et al. (bodybuilder (*n* = 15), sedentary (*n* = 15)). This resulted in a larger bodybuilder group (*n* = 31) and a control sedentary group (*n* = 15). In this process, data on three bodybuilders were excluded (part 1-PRE12, PRE14/citation-KY0006) because did not meet the RDA standards. A total of 28 bodybuilders were in the final bodybuilder group. Section 2.5 provides details on the characteristics of these groups. We then performed nutrient intake analysis.

### 2.3. Gut Microbiota

Fecal samples were collected from all subjects before and after supplement intake. Samples were stored in a deep freezer at −80 °C until use. Metagenomic DNA was separated using the FastDNA SPIN Kit for Soil (MP Biomedicals). Extracted DNA was placed on ice to maintain sample status.

After the isolated metagenomic DNA passed the quality control (QC) check, PCR amplification was performed using the 16S rRNA gene as a phylogenetic marker, with a fusion primer containing a barcode. The amplified product measured approximately 500–700 bp in size. The variable regions V1 to V3 in the 16S rRNA gene were identified in bacteria. Since bacteria belonging to the *Bifidobacterium* genus cannot be amplified with a general universal primer, 10% of a primer specific to bifidobacterium was used to perform PCR amplification. Subsequently, the thermal data were clustered with CD-Hit and UCLUST using the EzTaxon database, which organizes the 16S RNA gene sequences of standard strains and non-cultivated microorganisms. Next, ChunLab′s bioinformatics cloud platform, EZBiOCloud, was used to analyze alpha and beta diversity expressed in OTU, Chao1, ACE, and Shannon.

### 2.4. SCFA

SCFA analysis was performed by freezing the fecal sample and subjecting it to gas chromatography-mass spectrometry (GC-MS) [25]. The sample (∼80 mg dry matter) was aliquoted into an H3PO4 tube containing SCFAs and centrifuged for 5 min at 20,000× *g* after vortexing. The supernatant was separated and diluted with isocaproic acid solution at a ratio of 1:1 up to 1mL. Gas chromatography was performed at 190 °C using a an Innowax 30 m × 530 µm × 1 µm capillary column to measure SCFA concentration.

### 2.5. Nutrient Intake Analysis

The nutritional intake of athletes performing resistance exercise (bodybuilders, *n* = 28) and sedentary subjects (*n* = 15) were surveyed on two days of the week and one day on the weekend. They were requested to record all diets’ kinnutrid and quantity. A nutrition questionnaire was used in this process, which was supplemented with one-on-one consultation with the principle investigator. A researcher with more than 3 years analytical experience have coded the submitted diet using Computer Aided Nutritional analysis program (CAN-Pro) ver. 5.0 (The Korean Nutrition Society, Korea) to analyze the total number of kilocalories per day and the content of macronutrients and micronutrients (Table 2).

According to KRDA, the proper protein intake is 7–20% of the daily energy intake, and it is recommended that the daily intake of dietary fiber is more than 25 g. Bodybuilders were classified into three groups according to nutritional intake status based on the analyzed data, as follows.

- Group 1. Bodybuilder–high protein and meager dietary fiber (*n* = 12);
- Group 2. Bodybuilder–high protein and proper dietary fiber (*n* = 10);
- Group 3. Bodybuilder–proper protein and meager dietary fiber (*n* = 6);
- Group 4. Sedentary (*n* = 15).

### 2.6. Statistical Analysis

All of the data were analyzed by SPSS 253 (SPSS, Chicago, IL, USA) and GraphPad Prism 8.4.3 (Graph Pad Software, Inc., California, CA, USA).

## 3. Results

### 3.1. Microbiome Analysis (Part 1)

**Figure 2:**
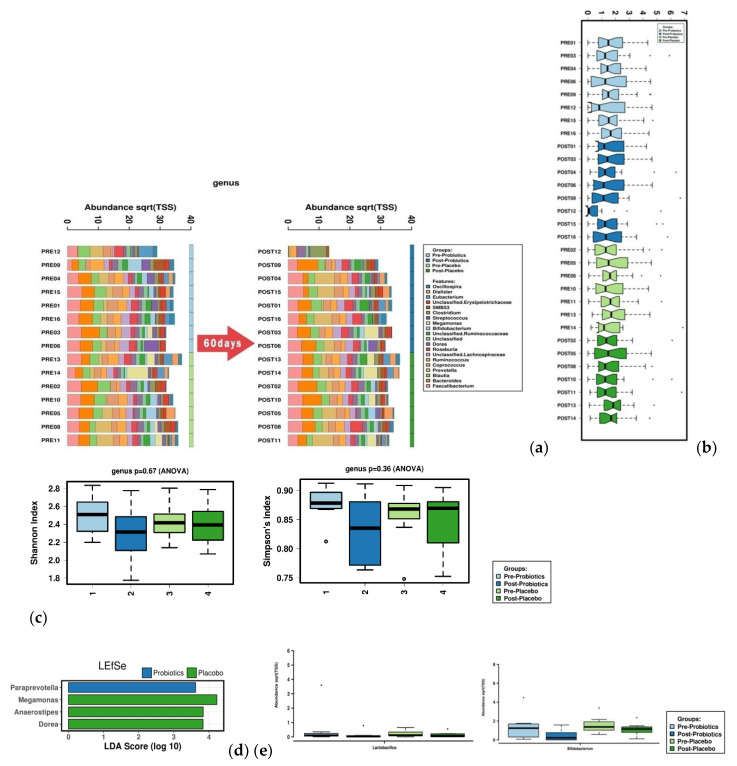
Microbiome analysis of groups treated with probiotics and placebo before and after supplement intake. (**a**) Changes in gut microbiome: Genus-level composition before and after 60 days of probiotic intake was analyzed by dividing the study population into probiotic intake and placebo intake groups. (**b**) The microbial composition of the probiotic and placebo groups before and after consumption was represented with BoxPlot. We stratified the sample and adjusted the opacity for clarity. (**c**) Alpha-diversity was measured with the Shannon Index and Simpson′s Index. No significant difference was observed between the probiotic and placebo groups before and after consumption. (**d**) The effect size (LEfSe) was measured via linear discriminant analysis (LDA). Based on pre–post intake and probiotic–placebo intake analysis, *Paraprevotella* in the probiotic group, along with *Megamonas*, *Anaerostipes*, and *Dorea* in the placebo group, were identified. (**e**) An analysis of *Lactobacillus* and *Bifidobacterium* species assessing changes in the abundance of four microorganisms (three *Lactobacilli* and one *Bifidobacterium*) used in the production of probiotics showed no significant differences between groups.

The groups exhibited enormous differences in protein and dietary fiber intake, as shown in Figure 2.

### 3.2. SCFAs (Part 1)

**Figure 3:**
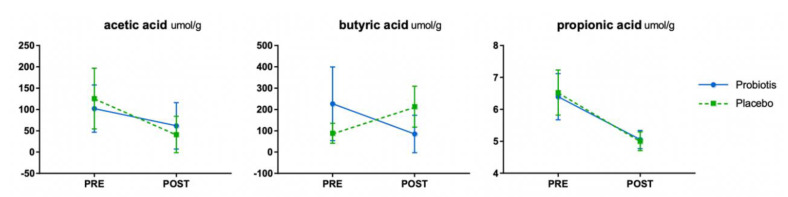
Short-chain fatty acids (SCFA) analysis. No significant difference was observed in acetic acid level before and after probiotics intake; however, both the intake and the control groups demonstrated a significant decrease over time. However, a difference in the level of butyric acid was observed before intake in the two groups. No significant difference was observed in the level of propionic acid, before or after consumption of probiotics.

There was no significant difference in the concentration of SCFAs, which are beneficial fermentation metabolites produced by intestinal microorganisms, in the probiotic and placebo groups (Figure 3).

### 3.3. Nutritional Intake Analysis (Part 2)

**Figure 4:**
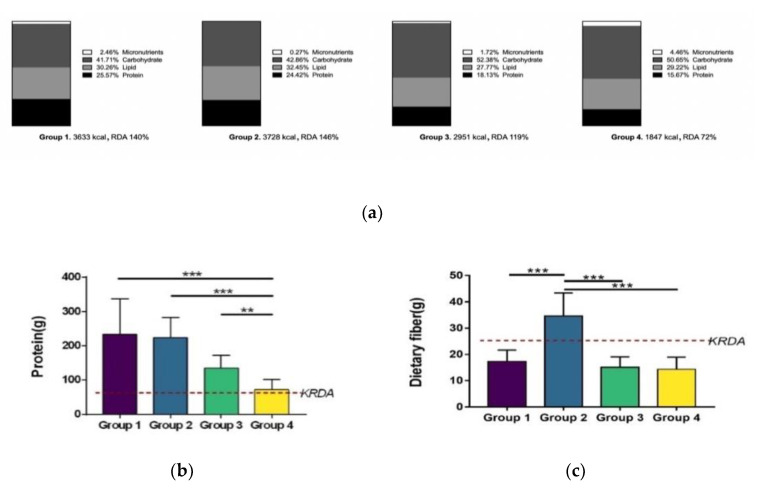
Nutritional intake analysis. (**a**) the proportion of carbohydrate, protein, fat, and dietary fiber in the total energy intake (TE) of each group is plotted. Protein intake decreased from group 1, which shows the most extreme nutritional intake, to group 4, which consumed a regular diet, with a concomitant increase in carbohydrate intake, to group 3. (**b**) Protein intake in each group met the recommended dietary intake. Groups 1, 2, and 3 showed significant differences compared to the sedentary group (*n* = 15). (Group1/Group4: *** *p* < 0.0005; Group2/Group4: *** *p* < 0.0005; Group3/Group4: ** *p* < 0.005). (**c**) Only the high protein and meager dietary fiber group met the recommended intake for dietary fiber, and there were significant differences compared with the other groups. (Group2/Group1: *** *p* < 0.0000; Group2/Group3: *** *p* < 0.0005; Group3/Group4: *** *p* < 0.0005).

The nutritional intake status of each group showed the largest difference in protein and dietary fiber based on group, as shown in Figure 4.

### 3.4. Microbiome Analysis (Part 2)

See Figure 5.

## 4. Discussion

According to a previous study [10,11,12], probiotic intake should have a significant influence on the gut microbiota of bodybuilders, but sometimes does not. We aimed to establish the effects of probiotic intake in subjects who exercise frequently and eat specific diets. Three forms of *Lactobacillus* and one form of *Bifidobacterium* were combined and administered to bodybuilders as a capsule. These four types of bacteria have been associated with exercise in prior studies. *L. acidophilus* not only inhibits the growth of *C. difficile* in the intestine by controlling the quorum-sensing signal and toxicity of the intestinal pathogen [14], but also improves intestinal inflammation by lowering Salmonella-induced NF-κB expression and the expression of the inflammatory cytokines TNF-α and IL-8 [20]. In addition, *L. casei* exhibits antioxidant and radical-scavenging properties [21] and strong anti-inflammatory and antioxidant effects, especially in combination with *L. acidophilus* [22,23]. A four-week intake of *B. bifidum* can also regulate the intestinal microbial ecosystem of healthy adults and increase the relative abundance of SCFA-producing bacteria [24]. Subjects who took supplements received pre-training on nutritional intake and were given the supplements for 60 days. They participated in a one-on-one mid-term follow-up conducted by the investigators. Sixty days of probiotic intake had no demonstrable effect on the gut microbiome and SCFAs of bodybuilders who were continuously exposed to exercise stimulation and consumed a highly nutritional diet. In contrast to previous studies, these results showed no improvements in the gut microbial environment following the consumption of probiotics. Of course, it is possible that too few subjects participated in this study and that they may not be representative of all bodybuilders, despite their similar diet and lifestyle habits and the fact that the study took place during bodybuilding season.

In this study, to elucidate the effects of nutritional intake/diet on the gut microbial environment and the effects of probiotic intake in bodybuilders, the subjects’ protein and dietary fiber intake was classified and compared with that of a control group with a sedentary lifestyle. In addition, based on the citation of the same athlete in the interpretation of the results, I tried to solve this weakness. Bodybuilders continuously undergo resistance training exercise, and due to the characteristics of their chosen sport, their protein intake is 2–4-fold higher than that of the general population. The correlation between nutritional intake and gut microbiome in these athletes confirmed that the intake of specific amounts of protein and dietary fiber influenced the composition and diversity of *Bifidobacterium* in the subjects. In this study, no significant differences were detected in digestive tract microbial diversity between Group 1 (high protein and reduced dietary fiber), which showed the most extreme nutritional intake, and Group 4 (Sedentary). In contrast, the gut microbial diversity index was higher than in Group 4 (Sedentary) when the protein intake (7–20% of daily energy intake) or dietary fiber intake (25 g) was close to the recommended amount, even if it represented extreme nutrition. Specifically, Group 2 (high protein and adequate dietary fiber), which met the recommended intake levels, showed a significantly higher microbial diversity index than Group 4 (Sedentary), despite the excessive protein intake. In the case of Group 3 (adequate protein and restricted dietary fiber), with a protein intake that was closer to the recommended amount, a larger number of microorganisms was found compared with Group 4 (Sedentary), even though dietary fiber intake was less than the recommended amount. Overall, based on gut microbiome analysis according to nutritional intake, we can conclude that probiotic supplementation without improvements in nutritional intake does not significantly affect the gut microbiome of bodybuilders.

Probiotic intake has recently been considered as a potential nutritional supplementation designed to promote the health of athletes [19]. Prior studies have consistently reported a positive association between exercise stimulation and gut microbiome health [13,14,15], and the effectiveness of probiotic intake has also been demonstrated in various studies [19]. This study, which used a combination of probiotic bacteria, showed superior nutritional and health benefits compared with exercise stimulation alone [26]. However, the associations between exercise and gut microbiome are not clearly established. For example, exposure to a high-fat diet increases the ratio of *Firmicutes/Bacteroidetes* and contributes to inflammation [27], while exercise similarly increases the ratio of *Firmicutes/Bacteroidetes* and decreases inflammation [27,28]. Consequently, several factors related to the interaction of nutrition and exercise remain unknown. If the subjects consume the same diet, designed by a nutritionist or dietarian with standard formula, the result will be more promising and innovative. Some studies have reported that specific kinds of dietary fiber intake can lead to various results [29,30]. Athletes in groups who share diet and exercise patterns can lead to advanced results on the correlation between exercise and gut microbiota.

The intestinal environment in the general population and among athletes differs depending on exercise type and eating habits. Therefore, to use probiotics as ergogenic aids, it is necessary to develop customized probiotics considering differences in gut microbiome according to exercise type based on nutritional intake. Further studies are needed on subjects who meet the general nutritional intake criteria in order to establish a definitive link between exercise and human microbiome health.

## 5. Conclusions

Generally, if bodybuilders with extreme nutritional intake patterns do not meet regular RDA criteria for either dietary fiber (more than 25 g intake) or protein (7–20% of daily energy intake), there is no significant difference in the diversity of the gut microbiome compared with the general population. In addition, the bodybuilders in this study demonstrated no positive effects even after consumption of probiotics, and ultimately showed no changes in SCFA levels. However, if protein and dietary fiber intake were in accordance with the recommended intake, the intestinal microbial diversity of bodybuilders was higher than that of the healthy general population, as shown in previous studies.

Importantly, especially for those who are involved in sports that require extreme nutrient intake, protein and dietary fiber intake were found to influence the gut microbiome. Therefore, in athletes, the intake of a balanced diet is essential to realize the benefits of exercise or probiotics. A follow-up study is necessary to optimize the guidelines for nutritional and probiotic intake, with an experimental design based on differences in the characteristics of different sports, dietary and cultural differences, and differences between individual probiotic species.

## Figures and Tables

**Figure 1:**
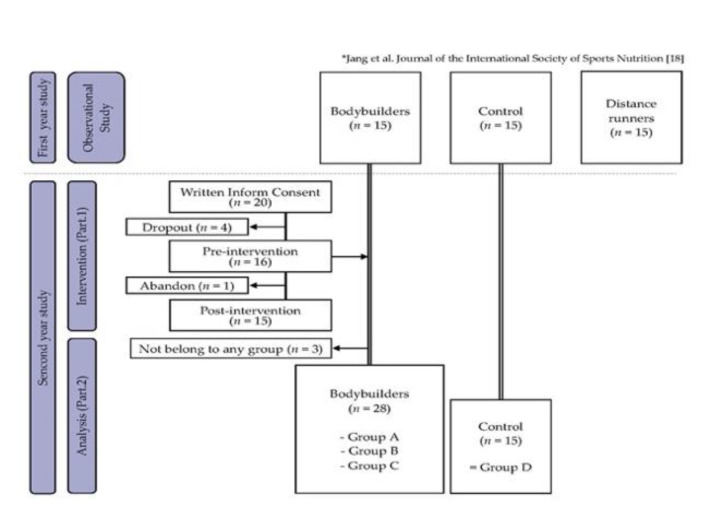
Study design for intervention study (Part 1, *n* = 15) and analysis study (Part 2, *n* = 43) [18].

**Figure 5:**
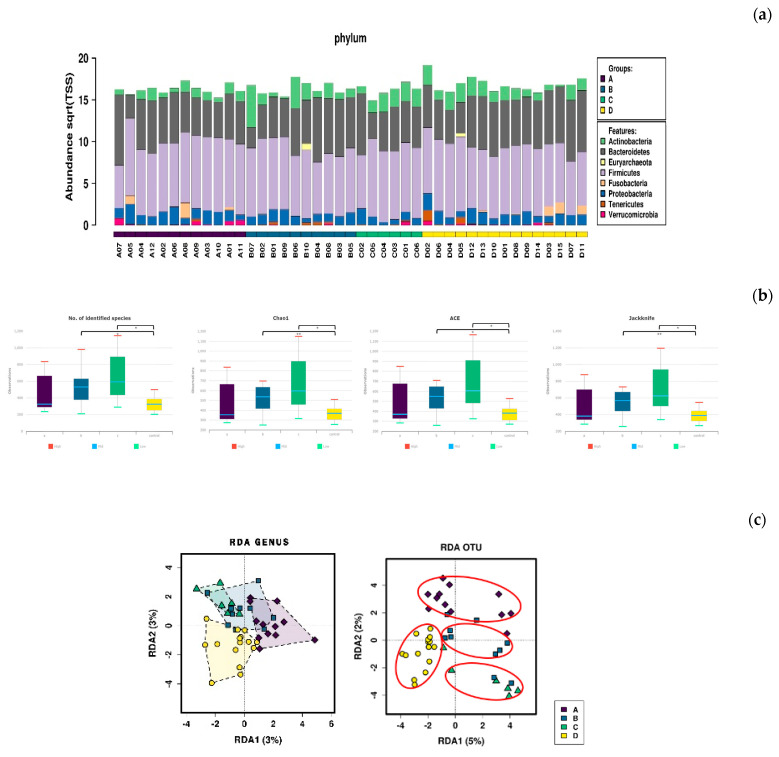

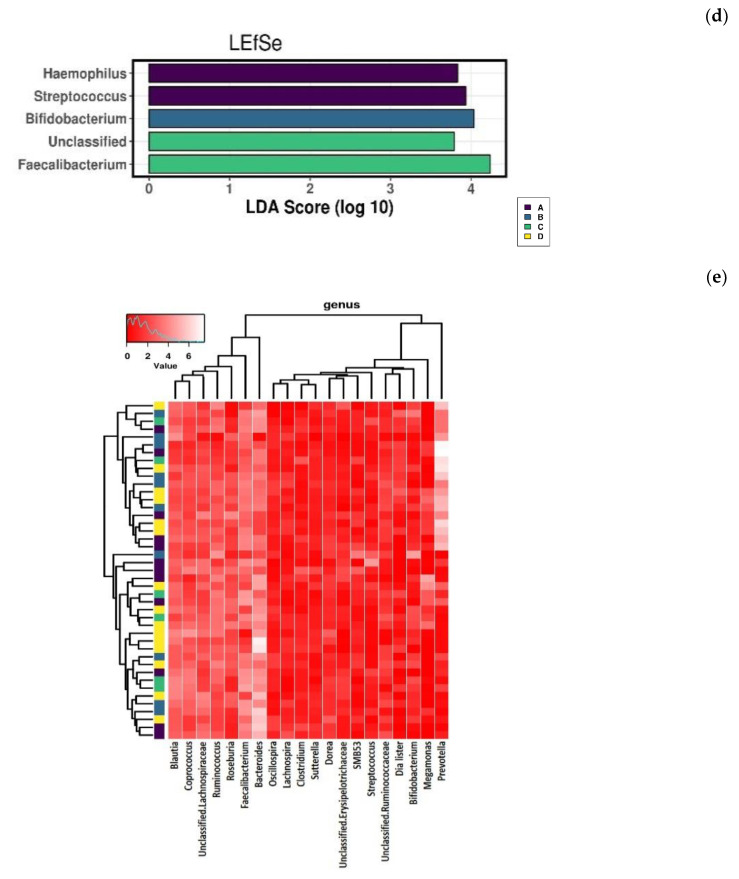

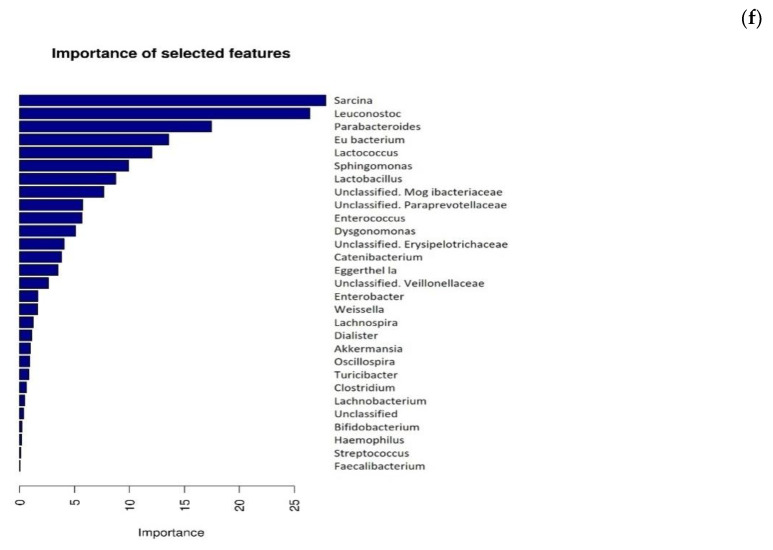
Microbiome analysis of each of the 4 groups according to nutritional intake (Part 2) (**a**) Phylum-level microbial composition analysis of the 3 groups of bodybuilders and the sedentary group. (**b**) Results of α-diversity analysis. Group 1 showed no significant differences compared with the sedentary group. Groups 2 and 3 showed significant differences compared to the sedentary group in the number of identified species (Group2/Group4: * *p* = 0.007; Group3/Group4: * *p* = 0.014), Chao1 (Group2/Group4: ** *p* = 0.004; Group3/Group4: * *p* < 0.011), ACE (Group2/Group4: * *p* = 0.007; Group3/Group4: * *p* = 0.018), and Jackknife (Group2/Group4: ** *p* = 0.004; Group3/Group4: * *p* = 0.011). (**c**) Redundancy analysis (RDA) showing separation between the groups in GENUS units and OUT (operational taxonomic units) units. (**d**) Effect size (LEfSe) was measured via LDA. The standard test for statistical significance was combined with additional tests for biological consistency and related effects to analyze taxonomic group, which was most likely to account for the differences between the groups at the genus level. *Fecalibacterium* was specified, in addition to Group 1: *Haemophilus*, *Streptococcus*; Group 2: *Bifidobacterium*; and Group 3: Unclassified bacteria. (**e**) The genus-level microbial patterns of the different groups of bodybuilders were visualized using a heat map. The individual samples in the groups are displayed. Color intensity was normalized to show the relative proportions of the four groups. The color range from white to red indicates the relative value (0–8) of all microorganisms. White color indicates that the genus is not detected or is not abundant, and deepening shades of red indicate increasing abundance. (**f**) Forest ranking based on average factor importance.

**Table 1:** Characteristics of the study subjects (intervention ver., Part 1).

	Probiotics (*n* = 8)	Placebo (*n* = 7)
Age (year)	26.50 ± 5.01	27.14 ± 5.93
Height (m)	1.75 ± 0.03	1.77 ± 0.05
Weigh (kg)	90.50 ± 9.34	83.58 ± 11.64
BMI (%)	29.71 ± 3.05	26.79 ± 3.32

Data are expressed as mean ± SD.

**Table 2:** Characteristics of the study subjects (nutrition analysis ver., Part 2).

	Group 1 (*n* = 12)	Group 2 (*n* = 10)	Group 3 (*n* = 6)	Group 4 (*n* = 15)
Age (years)	25.00 ± 3.05	25.90 ± 4.09	28.67 ± 6.12	26.27 ± 2.05
Height (m)	1.75 ± 0.05	1.75 ± 0.06	1.75 ± 0.04	1.76 ± 0.06
Weight (kg)	87.75 ± 9.42	87.05 ± 7.92	80.85 ± 11.56	79.82 ± 11.04
BMI (%)	28.52 ± 2.66	28.44 ± 2.55	26.51 ± 4.27	25.86 ± 4.18
Fat (kg)	11.71 ± 3.81	11.42 ± 4.41	11.27 ± 4.95	19.44 ± 7.90
Lean body mass (kg)	71.46 ± 9.37	72.38 ± 7.24	65.32 ± 8.24	56.53 ± 4.57
Percentage fat (%)	13.80 ± 4.68	12.92 ± 4.46	13.85 ± 4.93	23.95 ± 6.93

Data are expressed as mean ± SD in Table 2.

## Supplementary Materials

The following are available online at https://www.mdpi.com/2072-6643/12/10/2947/s1, Table S1: Random assignment list. Figure S1: 19 strains used probiotics in Korea.
