# Supplementary material for: The Effect of Athletes’ Probiotic Intake May Depend on Protein and Dietary Fiber Intake
Source: Nutrients. 2020 Sep 25;12(10):2947. doi: 10.3390/nu12102947 (PMC7650591; doi:10.3390/nu12102947)
Supplement: Supplementary file 1 [file nutrients-12-02947-s001.zip › Table S1.pdf]

The random assignment list		
Subjects	The group result	Notes
1	Probiotics	
2	Placebo	
3	Probiotics	
4	Probiotics	
5	Placebo	
6	Probiotics	
7	Placebo	*droped out
8	Placebo	
9	Probiotics	
10	Placebo	
11	Placebo	
12	Probiotics	
13	Placebo	
14	Placebo	
15	Probiotics	
16	Probiotics	
