# Supplementary figures and images for: The Effect of Athletes’ Probiotic Intake May Depend on Protein and Dietary Fiber Intake
Source: Nutrients. 2020 Sep 25;12(10):2947. doi: 10.3390/nu12102947 (PMC7650591; doi:10.3390/nu12102947)

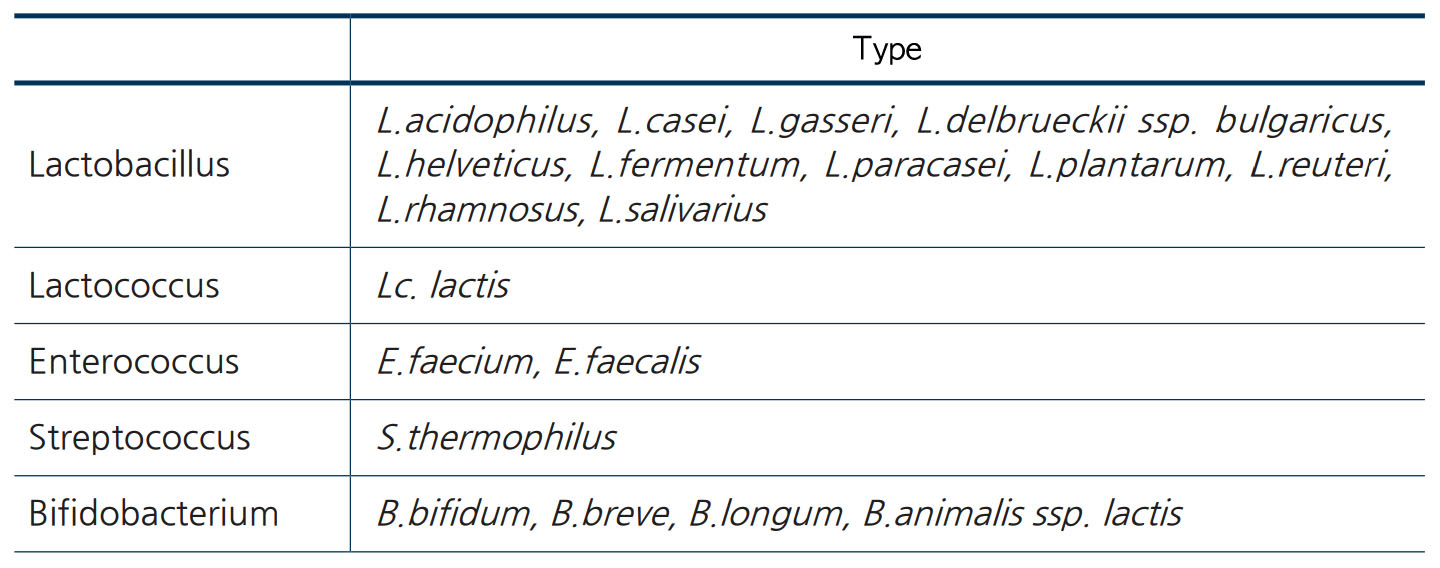

Supplement: Supplementary file 1 [file nutrients-12-02947-s001.zip › Figure S1.jpg]
